# Individual and contextual covariates of burnout: a cross-sectional nationwide study of French teachers

**DOI:** 10.1186/1471-2458-9-333

**Published:** 2009-09-10

**Authors:** Marie-Noël Vercambre, Pauline Brosselin, Fabien Gilbert, Eléna Nerrière, Viviane Kovess-Masféty

**Affiliations:** 1 Foundation for Public Health, MGEN, Paris, France; 2 EA4069, Université Paris Descartes, Paris, France

## Abstract

**Background:**

Limited information on the covariates of burnout syndrome in French teachers is available. The aim of this study was to evaluate the relative contributions of individual and contextual factors on the three burnout dimensions: emotional exhaustion, depersonalization, and reduced personal accomplishment.

**Methods:**

The source data come from an epidemiological postal survey on physical and mental health conducted in 2005 among 20,099 education workers (in activity or retired) selected at random from the health plan records of the national education system. The response rate was 52.4%. Teachers in activity currently giving classes to students who participated in the survey (n = 3,940) were invited to complete a self-administered questionnaire including the Maslach Burnout Inventory. 2,558 teachers provided complete data (64.9%). Variables associated with high emotional exhaustion (highest quartile of score), high depersonalization (highest quartile), and reduced personal accomplishment (lowest quartile) were evaluated using multivariate logistic regression. Studied variables referred to demographic characteristics, socio-professional environment, job dissatisfaction, experienced difficulties at work, and teaching motivations.

**Results:**

Different variables were associated with each burnout dimension. Female teachers were more susceptible to high emotional exhaustion and reduced personal accomplishment, whereas male teachers were more susceptible to high depersonalization. Elementary school teachers were more susceptible to high emotional exhaustion, but less susceptible to high depersonalization and reduced personal accomplishment than their higher school level counterparts. Experienced difficulties with pupils were associated with all three dimensions. A socio-economically underprivileged school neighbourhood was also related to high emotional exhaustion and high depersonalization.

**Conclusion:**

Programs to enhance teaching environment might be an interesting approach to try to prevent burnout. It would be useful to take the different dimensions into account in planning the intervention.

## Background

"Burnout" is a syndrome of emotional exhaustion, depersonalization, and reduced personal accomplishment which arises in response to chronic stress in jobs where individuals work with people [1]. Emotional exhaustion refers to feelings of being emotionally overextended and drained by others. Depersonalization refers to development of dehumanized and cynical attitudes toward people who are recipients of one's services. Reduced personal accomplishment refers to a decline in one's feeling of competence and successful achievement in work. Despite increasing interest in burnout in a wide range of occupations [2,3], the burnout phenomenon has been studied principally in human service professions [4]. In educational settings, interest is growing because the teaching profession has dramatically evolved over recent years, with teaching now demanding more time and energy, but with fewer resources available and a lack of social recognition [5]. In this context, variables associated with burnout in teachers have been studied extensively [6].

Certain background variables have been put forward as potential risk factors for burnout, including gender, age, marital status and grade level taught. Male teachers have been shown to be more susceptible to depersonalization than females [7,8], whereas investigations of the influence of age have yielded more inconsistent findings. On the whole, age seems to be inversely correlated with emotional exhaustion [9,10]. With respect to grade level taught, intermediate and high school teachers were more likely to exhibit a higher degree of depersonalization than their elementary school counterparts [8,9].

In addition to background factors, several organizational and personality factors have been related to burnout. Concerning organizational factors, recent investigations have indicated that role conflict, role ambiguity, imbalance of effort and reward, as well as perceptions of job stressors, were important factors in teacher burnout. In particular, the number of pupils [10], time constraints [11], and work overload [12] have been related to a high degree of emotional exhaustion. Erosion of the classroom climate also appears to have an impact on stress and subsequent burnout. Notably, pupil disrespect was associated with a higher degree of emotional exhaustion and depersonalization, whereas pupil lack of sociability was associated with a higher degree of depersonalization and a lower degree of personal accomplishment [13]. Moreover, lack of support (as evaluated through a general measure of social support, including items on emotional support from colleagues and staff members) has been associated with a higher degree of emotional exhaustion and depersonalization [14]. On the whole, job dissatisfaction was closely related to feelings of burnout [15] on all three dimensions. Lastly, individual resources and personality factors have been studied in teacher samples to explain why individuals in the same work environment and having the same educational and experience backgrounds often respond differently in terms of burnout. Notably, neuroticism (which corresponds broadly to individual susceptibility to psychological distress and inability to cope with stress) has been related to higher emotional exhaustion [4,11,16]. Other studies have related sense of efficacy [17-19], perception of superiority [20], and hardiness [21] to burnout syndrome.

Overall, the literature indicates that individual characteristics and contextual factors are closely interwoven in the development of burnout in teacher. Nonetheless, findings have been somewhat inconsistent between studies, possibly due, at least in part, to a failure to take into account potential confounding factors adequately in the statistical analysis.

The goal of the present study was to evaluate potential independent associations between a variety of personal and contextual factors and burnout using multivariate techniques. We also wished to test the hypothesis that the association patterns differ for each of the three burnout dimensions, and thereby evaluate the multidimensionality of the burnout syndrome [22]. The choice of individual and contextual variables to evaluate as potential covariates of burnout was inspired by the above literature. To be considered, these should have been sufficiently well documented in the questionnaire used. We also considered some factors that had, to our knowledge, never been studied in relation to burnout, namely the school neighbourhood and the reasons why the teaching profession was chosen. School neighbourhood is a proxy that evaluates neighbourhood-level psychosocial stressors such as experience of incivility, drug misuse, youngsters frequently hanging around, rubbish on the streets, feeling unsafe or dissatisfaction with the quality of green space. In the general population, these stressors have been related to level of perceived stress [23], as well as self-reported health [24]. Our hypothesis was that teachers in socio-economically underprivileged school neighbourhood will be more exposed to stressful situations and consequently, more susceptible to burnout. Regarding teaching motivations, idealism in teachers could be both a protective factor and a risk factor with respect to certain psychological symptoms. Indeed, idealist teachers are probably more psychologically involved at school, because of their concern toward pupil success. Their mental wellness will then depend on their perceptions that reality and ideals go together well or not [25]. We hypothesized that teachers who declared to have chosen teaching because they felt a calling for that (such as a "desire to make young people grow up") were less susceptible to depersonalization but more susceptible to emotional exhaustion than those teachers who declared to have chosen teaching for job security.

In order to test these various hypotheses, we analyzed recent data from a large sample of policyholders of the "Mutuelle Générale de l'Éducation Nationale" (MGEN). This health care insurance company covers everyone currently working or who has previously worked within the public education system in France.

## Methods

### Study population and selection process

The population of the source epidemiological survey was a sample of 20,099 insurance policyholders, aged 18 years or over, selected at random (one in every hundred) from a register of unique policy numbers assigned to individual affiliated members of the MGEN. Of the 20,099 selected policyholders, 49 could not be contacted because they had died, and 411 because their address was incorrect. In January 2005, the 19,639 remaining individuals received a questionnaire by mail, accompanied by a letter explaining that the survey participation was voluntary and that anonymity was ensured through the use of identifying numbers. As required by the observational research regulation in France, this study was approved by both the national authorities responsible for protecting privacy and personal data: the "Comité Consultatif sur le Traitement de l'Information en matière de Recherche dans le domaine de la Santé" (CCTIRS) and the "Commission Nationale de l'Informatique et des Libertés" (CNIL). The global response rate after two consecutive mail shots was 52.4% (i.e. 10,288 respondents).

The present study was conducted among teachers in active employment who were currently giving classes to students. These were precisely invited to complete a specific part including a questionnaire on burnout. Of the 10,288 respondents, 6,532 were teachers, of whom 3,940 individuals were currently teaching. A further 1,382 individuals were excluded due to missing data for the burnout questionnaire (n = 756) or for the covariates of interest (n = 626). The final study sample thus included 2,558 working teachers (64.9%) with complete data for all variables studied.

### Characteristics of the study sample

Our study sample reflected the proportion of men and women in the national population of teachers within the public education system (64.7% women vs. 63.8% in the French public system [26]) but differed slightly with respect to age and grade level taught. Teachers aged less than 30 were under-represented (8% vs. 16%) and those aged 50 or more were over-represented (37% vs. 29%). There were also fewer university teachers than expected (7.1% vs. 9.3%). In our sample, gender was closely associated with other variables of interest (Table 1), in particular, with grade level taught, with nursery and primary teachers being predominately female, and secondary school and university teachers predominantly male.

**Table 1:** Description of the study sample by gender

	**Men** **n = 902** **%**	**Women** **n = 1,656** **%**	**Total** **n = 2,558** **%**	**p**
Gender	35.3	64.7	100	
				
Age				< 0.01
< 30 years	8.8	7.2	7.7	
30 to 39 years	24.7	30.9	28.7	
40 to 49 years	25.4	28.0	27.1	
≥50 years	41.1	33.9	36.5	
				
Marital status				< 0.01
Single	22.7	24.5	23.9	
In couple	69.3	60.6	63.6	
Previously married	8.0	14.9	12.5	
				
Grade level taught				< 0.01
Nursery school	2.8	19.0	13.3	
Primary school	18.9	27.1	24.2	
Early secondary school	25.6	25.7	25.7	
Secondary school	23.7	14.7	17.9	
Vocational school	12.5	6.8	8.8	
University	12.1	4.4	7.1	
Special classes for pupils with learning disabilities	4.4	2.4	3.1	
				
Teaching social neighbourhood†				0.45
Normal	81.4	80.1	80.6	
Underprivileged	18.6	19.9	19.4	

* Chi^2 ^test

† As defined by the French Ministry of Education

### Measurement of burnout

The French version of the Maslach Burnout Inventory [1] adapted to education settings (MBI-ES [27]) was used to evaluate burnout syndrome among teachers participating in the study.

The MBI is nowadays the most widely used instrument to measure burnout in individuals [2]. The 22-item version of the MBI consists of nine items that measure emotional exhaustion, five items that measure depersonalization, and eight items that measure personal accomplishment. Each item can be answered on a 7-point Likert scale ranging from "never" (= 0) to "daily" (= 6). High scores on emotional exhaustion and depersonalization and low scores on personal accomplishment are indicative of burnout.

The general version of the MBI was validated in French in two samples of day-care workers and nurses in Quebec [28]. The reliability and validity of the instrument has also been confirmed in a sample of French teachers [29]. In the current study, Cronbach's alpha coefficients were 0.87 for the emotional exhaustion subscale, 0.61 for depersonalization, and 0.82 for personal accomplishment.

### Outcomes of interest

Maslach and Jackson originally considered burnout subscale scores as continuous variables which could be expressed as a degree of severity ranging from low to medium to high [1]. They did not define a clinical threshold that indicated the presence or absence of burnout. In contrast, others have conceptualized these scores as dichotomous, in order to distinguish individuals with serious burnout symptoms from others. To this end, cut-off scores corresponding to burnout diagnosis (when available) [14], or to the extreme quartile of MBI scores have been proposed [30]. For the purposes of this study, we adapted this latter approach. Emotional exhaustion and depersonalization were respectively assigned a value of '1' (presence of burnout symptom) when scores were above the 3^rd ^quartile, whereas personal accomplishment was assigned a value of '1' if the score was below the 1^st ^quartile. A major advantage of this dichotomous approach based on the extreme quartile is that it eliminates the problem of potentially small sample sizes for case groups and permits subsequent use of logistic regression models to test associations between burnout and variables of interest. Such models are well adapted to this purpose, since no *a priori *hypotheses are needed concerning 'normality' and homogeneity of variance, and because non linear phenomena can be detected.

Each dichotomous outcome (MBI subscale score assignment) was modelled independently, in order to preserve the multidimensional evaluation of burnout. Indeed, these three conceptually distinct burnout dimensions have been confirmed empirically in populations of teachers through both factor analysis and internal consistency studies [31-33].

### Description of study variables

We regrouped the potential covariates of burnout studied into three categories, corresponding to demographic characteristics, socio-professional environment and other work-related factors.

The first category comprised gender, age and marital status. Age was divided into four groups: < 30 years; 30-39 years; 40-49 years; ≥50 years; marital status was divided into three groups: single; in couple; previously married (widowed, divorced, or separated).

The socio-professional environment was described through the grade level, the teaching social neighbourhood, and the number of pupils. Grade level was divided into seven categories: nursery school; primary school; early secondary school; secondary school; university; vocational school; special classes for pupils with learning disabilities. Social school neighbourhood was appraised through a dichotomous variable indicating if or not the school was localized in an "educational priority area", a definition of socioeconomically underprivileged areas introduced by the French Ministry of Education in 1981. This notion was used to guide education policies aimed at correcting social, economic and cultural inequality by concentrating educational investment in areas where student failure was highest. Concerning the number of pupils, a three-category variable (low, in the norm, high) was constructed depending on the median class size observed in the corresponding grade level: number of pupils less than (the median - 2); comprised in the median ± 2 range; greater than (the median + 2).

Other work-related variables dealt with reported dissatisfaction with certain job aspects (timetable, time constraints, salary, resources, training), experienced difficulties with others (pupils, colleagues, parents, administration), as well as reasons for choosing the teaching profession (job security, feeling a calling for teaching). Each of these variables was investigated in the self administered questionnaire in a yes/no mode and coded dichotomously.

### Statistical analysis

In a first step, we described the distribution of the scores obtained in the study sample for each of the three burnout subscales and compared mean scores obtained with other similar studies of teachers performed elsewhere in Europe.

In a second step, for each of the three burnout dimensions, we compared frequency distributions of the variables of interest between subjects assigned to the burnout group (according to extreme score quartile) and the non-burnout reference group.

Finally, we related the probability of being in the burnout group with the variables of interest using logistic regression models, initially considering each variable separately to evaluate the unadjusted association with the burnout dimension (bivariate analysis) and subsequently introducing all variables into a multivariate model in order to evaluate potentially independent associations when adjusting for confounders. The strength of the associations identified are presented as odds ratios together with their 95 percent confidence intervals (OR [95%CI]).

All analyses were performed using Stata SE 9 software. A probability threshold of 0.05 was considered statistically significant and all statistical tests were two sided.

## Results

### Descriptive analysis of burnout dimension scores

Table 2 presents the scores observed in the study sample for the three dimensions of burnout. Mean scores (standard deviations) were 18.1 (10.2) for emotional exhaustion, 3.3 (3.7) for depersonalization, and 31.2 (8.6) for personal accomplishment. Table 3 presents comparable values in Europe that were picked up from the literature [13-15,29,34-41].

**Table 2:** Scores on the three burnout dimensions in the study sample

	**Emotional Exhaustion**				**Depersonalization**				**Personal Accomplishment**			
n	mean (sd)	q1	median	q3	mean (sd)	q1	median	q3	mean (sd)	q1	mean	q3
2,558	18.1 (10.2)	10	17	24	3.3 (3.7)	1	2	5	31.2 (8.6)	26	32	38

sd: standard deviation; q1: first quartile; q3: third quartile

**Table 3:** Scores on the three burnout dimensions: comparison with other studies of teachers in Europe

**N**	**Population**	**Localization**	**Reference**	**Emotional exhaustion** **mean (sd)**	**Depersonalization** **mean (sd)**	**Personal accomplishment** **mean (sd)**
2,258	Teachers	France	Present study	18.1 (10.2)	3.3 (3.7)	31.2 (8.6)
100	Primary school teachers	United Kingdom	[13]	23.4 (10.3)	4.6 (4.3)	37.2 (6.4)
365	Mathematics teachers	Netherlands	[14]	14.0 (8.1)	5.6 (3.5)	27.3 (6.4)
100	Primary and secondary school teachers	Greece	[15]	15.0 (8.4)	3.9 (3.4)	39.7 (5.4)
217	Teachers	France	[29]	15.0*	4.5*	30.6*
299	Representative sample of teachers	Italy	[29]	18.5*	3.1*	33.9*
304	Secondary school teachers	Netherlands	[34]	18.2 (1.2)	5.6 (5.4)	34.7 (7.4)
215	id	Greece	[35]	14.5 (10.3)	2.9 (3.7)	37.4 (8.0)
169	id	Italy	[36]	20.0 (13.1)	2.3 (3.7)	35.8 (8.1)
198	id	Spain	[37]	21.2 (11.1)	3.6 (4.1)	31.9 (9.2)
297	id	Germany	[38]	18.5 (9.6)	4.2 (4.4)	32.5 (8.0)
232	id	Finland	[39]	19.5 (10.6)	3.6 (3.9)	32.8 (8.0)
166	id	United Kingdom	[40]	28.5 (11.9)	8.6 (6.6)	29.0 (8.5)
128	id	Belgium	[41]	20.4 (13.3)	4.2 (4.1)	31.3 (9.2)

*Standard deviation not available

sd: standard deviation

### Bivariate analysis

In the unadjusted analysis step (Table 4), each of the burnout dimensions was associated with a specific set of covariates. The sets of variables associated with emotional exhaustion and with depersonalization were quite similar, although for two variables, gender and grade level taught, the associations were in the opposite directions for these two dimensions. In contrast, the set of variables associated with reduced personal accomplishment contained a smaller number of quite different variables.

**Table 4:** Bivariate associations with each of the three burnout dimensions: odds ratios and their 95% confidence intervals for demographic characteristics, socio-professional environment and work-related factors

	**Emotional exhaustion**					**Depersonalization**					**Personal accomplishment**				
	**Ref**	High*	**Probability of high score†**			**Ref**	High*	**Probability of high score†**			**Ref**	Low*	**Probability of low score†**		
	**%**	**%**	**OR**	**95%CI**	**p**	**%**	**%**	**OR**	**95%CI**	**p**	**%**	**%**	**OR**	**95%CI**	**p**
**Demographic characteristics**															
Gender															
Men	38.1	26.6	1			32.6	45.6	1			36.0	33.1	1		
Women	61.9	73.4	1.7	1.4-2.1	< 0.01	67.4	54.4	0.6	0.5-0.7	< 0.01	64.0	66.9	1.1	0.9-1.4	0.18
Age															
< 30 years	7.8	7.7	1.1	0.8-1.6	0.56	7.5	8.7	1.3	0.9-1.9	0.17	8.4	5.7	0.7	0.5-1.1	0.10
30 to 39 years	28.8	28.5	1.1	0.9-1.4	0.38	28.3	30.4	1.2	0.9-1.6	0.16	29.0	28.0	1.0	0.8-1.3	0.82
40 to 49 years	27.8	24.6	1			27.7	24.6	1			27.5	25.8	1		
≥50 years	35.6	39.2	1.2	1.0-1.6	0.06	36.5	36.4	1.1	0.9-1.4	0.37	35.1	40.5	1.2	1.0-1.5	0.08
Marital status															
Single	23.3	25.6	1.2	1.0-1.5	0.12	22.9	27.6	1.3	1.0-1.6	0.02	22.9	26.9	1.3	1.0-1.5	0.04
Couple	64.8	60.0	1			64.7	59.6	1			64.6	60.8	1		
Previously married	11.9	14.4	1.3	1.0-1.7	0.05	12.4	12.8	1.1	0.8-1.5	0.46	12.5	12.3	1.0	0.8-1.4	0.78
															
**Socioprofessional environment**															
Grade level taught															
Nursery school	12.5	15.8	0.9	0.7-1.2	0.60	14.4	8.9	0.8	0.5-1.1	0.20	13.9	11.3	1.3	0.9-1.8	0.16
Primary school	22.1	30.4	1			25.2	19.9	1			26.5	17.0	1		
Early secondary school	25.4	26.4	0.8	0.6-1.0	0.03	23.7	33.7	1.8	1.4-2.4	< 0.01	26.1	24.6	1.5	1.1-1.9	< 0.01
Secondary school	19.6	12.6	0.5	0.3-0.6	< 0.01	17.6	18.9	1.4	1.0-1.9	0.05	15.5	25.2	2.5	1.9-3.4	< 0.01
Vocational school	9.3	7.5	0.6	0.4-0.9	< 0.01	9.2	7.5	1.0	0.7-1.6	0.84	8.1	11.0	2.1	1.5-3.0	< 0.01
University	8.2	3.5	0.3	0.2-0.5	< 0.01	7.1	6.8	1.2	0.8-1.8	0.40	6.2	9.8	2.5	1.7-3.6	< 0.01
Special classes for pupils with learning disabilities	2.9	3.7	0.9	0.5-1.5	0.78	2.8	4.3	1.9	1.1-3.3	0.02	3.7	1.1	0.5	0.2-1.0	0.06
Teaching social neighbourhood ‡															
Normal	82.4	74.9	1			82.1	74.7	1			79.8	82.8	1		
Underprivileged	17.6	25.1	1.6	1.3-1.9	< 0.01	17.9	25.3	1.6	1.2-1.9	< 0.01	20.2	17.2	0.8	0.6-1.0	0.10
Number of pupils taught §															
Low	27.3	21.1	0.7	0.6-0.9	< 0.01	25.9	25.1	0.9	0.8-1.2	0.66	25.0	28.2	1.2	1.0-1.5	0.11
In the norm	52.5	55.5	1			53.0	54.2	1			53.9	51.2	1		
High	20.3	23.4	1.1	0.9-1.4	0.46	21.1	20.7	1.0	0.7-1.2	0.74	21.1	20.6	1.0	0.8-1.3	0.83
															
**Work-related factors**															
Satisfied with time constraints	93.3	84.6	1			92.2	87.2	1			91.4	90.6	1		
Unsatisfied	6.7	15.4	2.5	1.9-3.3	< 0.01	7.8	12.8	1.7	1.3-2.3	< 0.01	8.6	9.4	1.1	0.8-1.5	0.53
Satisfied with time table	88.6	79.8	1			87.6	81.8	1			86.8	85.4	1		
Unsatisfied	11.4	20.2	2.0	1.5-2.5	< 0.01	12.4	18.2	1.6	1.2-2.0	< 0.01	13.2	14.6	1.1	0.9-1.5	0.36
Satisfied with salary	52.2	35.5	1			49.5	42.9	1			47.4	50.6	1		
Unsatisfied	47.8	64.5	2.0	1.6-2.4	< 0.01	50.5	57.1	1.3	1.1-1.6	< 0.01	52.6	49.4	0.9	0.7-1.1	0.17
Satisfied with resources	50.6	36.3	1			48.7	40.8	1			47.3	46.5	1		
Unsatisfied	49.4	63.7	1.8	1.5-2.2	< 0.01	51.3	59.2	1.4	1.1-1.7	< 0.01	52.7	53.5	1.0	0.9-1.2	0.71
Satisfied with training	40.8	30.6	1			39.3	34.0	1			39.0	36.2	1		
Unsatisfied	59.2	69.4	1.6	1.3-1.9	< 0.01	60.7	66.0	1.3	1.0-1.5	0.03	61.0	63.8	1.1	0.9-1.4	0.22
															
Have chosen to be teacher for the job security	38.8	47.5	1.4	1.2-1.7	< 0.01	39.3	47.4	1.4	1.1-1.7	< 0.01	40.6	42.0	1.1	0.9-1.3	0.51
Not for this reason particularly	61.2	52.5	1			60.7	52.6	1			59.4	58.0	1		
Have chosen to be teacher because felt a calling for that	33.1	34.6	1.1	0.9-1.3	0.50	34.3	30.0	0.8	0.7-1.0	0.06	37.1	22.4	0.5	0.4-0.6	< 0.01
Not for this reason particularly	66.9	65.4	1			65.7	70.0	1			62.9	77.6	1		
No difficulties with pupils	61.8	44.2	1			61.2	42.6	1			60.0	49.9	1		
Difficulties	38.2	55.8	2.0	1.7-2.5	< 0.01	38.8	57.4	2.1	1.8-2.6	< 0.01	40.0	50.1	1.5	1.3-1.8	< 0.01
No Difficulties with parents	84.9	72.2	1			82.8	77.8	1			82.9	78.4	1		
Difficulties	15.1	27.8	2.2	1.7-2.7	< 0.01	17.2	22.2	1.4	1.1-1.7	< 0.01	17.1	21.6	1.3	1.1-1.7	0.01
No difficulties with Colleagues	88.2	79.0	1			86.6	83.2	1			86.2	85.0	1		
Difficulties	11.8	21.0	2.0	1.6-2.5	< 0.01	13.4	16.8	1.3	1.0-1.7	0.04	13.8	15.0	1.1	0.9-1.4	0.46
No difficulties with the administration	85.1	73.6	1			84.7	72.7	1			83.1	80.0	1		
Difficulties	14.9	26.4	2.0	1.6-2.5	< 0.01	15.3	27.3	2.1	1.7-2.6	< 0.01	16.9	20.0	1.2	1.0-1.5	0.08

OR: odds ratio; 95%CI: 95 percent confidence interval

* 'High score' means 'in the highest quartile of the scores observed in the sample', 'low score' means 'in the lowest quartile'

† Probability modelled through an univariate logistic regression analysis

‡ As defined by the French Ministry of Education

§ The number of pupils taught was divided into three groups depending on the median class size observed in the corresponding grade level taught: number of pupils taught less than (the median - 2); comprised in the median ± 2 range; greater than (the median + 2)

### Multivariate analysis

In the fully-adjusted logistic regression model (Table 5), a high score on the emotional exhaustion subscale was significantly associated with female gender, age ≥50 years, teaching in a nursery or a primary school, teaching in an underprivileged neighbourhood, teaching big classes, as well as reporting dissatisfaction with time constraints, timetable, salary and resources; having chosen the teaching profession for job security; and complaining of difficulties with pupils, colleagues or administration.

**Table 5:** Multivariate associations with each of the three burnout dimensions: odds ratios and their 95% confidence intervals for demographic characteristics, socio-professional environment and work-related factors

	**Emotional exhaustion** High score*†			**Depersonalization** High score*†			**Personal accomplishment** Low score*†		
	**OR**	**95% CI**	**p**	**OR**	**95% CI**	**p**	**OR**	**95% CI**	**p**
**Demographic characteristics**									
Gender									
Men	1			1			1		
Women	1.6	1.3-2.0	< 0.01	0.6	0.5-0.7	< 0.01	1.4	1.1-1.7	< 0.01
Age									
< 30 years	1.4	0.9-2.1	0.10	1.1	0.7-1.7	0.54	0.7	0.5-1.1	0.14
30 to 39 years	1.0	0.8-1.4	0.73	1.0	0.8-1.4	0.75	1.0	0.7-1.2	0.70
40 to 49 years	1			1			1		
≥50 years	1.7	1.3-2.1	< 0.01	1.1	0.8-1.4	0.62	1.2	0.9-1.5	0.13
Marital status									
Single	1.2	0.9-1.5	0.17	1.3	1.0-1.6	0.05	1.4	1.1-1.7	< 0.01
Couple	1			1			1		
Previously married	1.1	0.8-1.4	0.68	1.1	0.8-1.6	0.43	1.0	0.7-1.3	0.89
**Socioprofessional environment**									
Grade level taught									
Nursery school	0.8	0.6-1.1	0.21	0.9	0.6-1.3	0.46	1.1	0.8-1.6	0.46
Primary school	1			1			1		
Early secondary school	0.7	0.5-0.9	< 0.01	1.7	1.2-2.2	< 0.01	1.5	1.1-2.0	< 0.01
Secondary school	0.6	0.4-0.8	< 0.01	1.4	1.0-2.0	0.03	2.9	2.1-4.0	< 0.01
Vocational school	0.8	0.5-1.1	0.17	1.1	0.7-1.7	0.74	2.6	1.7-3.8	< 0.01
University	0.4	0.3-0.7	< 0.01	1.5	0.9-2.4	0.11	3.4	2.2-5.2	< 0.01
Special classes for pupils with learning disabilities	0.8	0.5-1.4	0.44	1.5	0.9-2.8	0.14	0.6	0.2-1.3	0.17
Teaching social neighbourhood ‡									
Normal	1			1			1		
Underprivileged	1.4	1.1-1.8	< 0.01	1.5	1.1-1.9	< 0.01	1.0	0.7-1.2	0.77
Number of pupils taught §									
Low	1			1			1		
In the norm	0.8	0.6-1.0	0.07	0.9	0.7-1.2	0.48	1.1	0.9-1.3	0.54
High	1.3	1.0-1.7	0.05	1.1	0.8-1.4	0.71	0.9	0.7-1.2	0.40
**Work-related factors**									
Satisfied with time constraints	1			1			1		
Unsatisfied	1.5	1.1-2.2	0.01	1.2	0.9-1.8	0.25	1.1	0.8-1.6	0.65
Satisfied with time table	1			1			1		
Unsatisfied	1.5	1.1-2.0	< 0.01	1.2	0.9-1.7	0.17	1.0	0.8-1.4	0.86
Satisfied with salary	1			1			1		
Unsatisfied	1.7	1.4-2.1	< 0.01	1.1	0.9-1.4	0.21	0.9	0.7-1.1	0.23
Satisfied with resources	1			1			1		
Unsatisfied	1.4	1.1-1.7	< 0.01	1.2	1.0-1.5	0.08	1.1	0.9-1.3	0.61
Satisfied with training	1			1			1		
Unsatisfied	1.2	0.9-1.4	0.20	1.3	1.0-1.6	0.05	1.3	1.0-1.6	0.02
Have chosen to be teacher for the job security	1.4	1.1-1.7	< 0.01	1.3	1.1-1.6	0.01	1.0	0.8-1.2	0.87
Not for this reason particularly	1			1			1		
Have chosen to be teacher because felt a calling for that	1.0	0.8-1.3	0.84	0.8	0.7-1.0	0.10	0.5	0.4-0.6	< 0.01
Not for this reason particularly	1			1			1		
No difficulties with pupils	1			1			1		
Difficulties	1.7	1.3-2.0	< 0.01	1.7	1.4-2.1	< 0.01	1.5	1.2-1.9	< 0.01
No Difficulties with parents	1			1			1		
Difficulties	1.2	0.9-1.6	0.15	1.2	0.9-1.5	0.28	1.5	1.1-1.9	< 0.01
No difficulties with Colleagues	1			1			1		
Difficulties	1.4	1.1-1.9	0.01	1.1	0.8-1.5	0.61	1.0	0.8-1.4	0.89
No difficulties with the administration	1			1			1		
Difficulties	1.6	1.3-2.1	< 0.01	1.6	1.2-2.0	< 0.01	1.0	0.8-1.3	0.82

OR: odds ratio; 95%CI: 95 percent confidence interval

* 'High score' means 'in the highest quartile of the scores observed in the sample', 'low score' means 'in the lowest quartile'

† Probability modelled through a full logistic regression analysis including simultaneously all variables listed above

‡ As defined by the French Ministry of Education

§ The number of pupils taught was divided into three groups depending on the median class size observed in the corresponding grade level taught: number of pupils taught less than (the median - 2); comprised in the median ± 2 range; greater than (the median + 2)

A high score on the depersonalization subscale was significantly associated with male gender, being single, teaching in an early secondary or a secondary school, teaching in an underprivileged neighbourhood, as well as reporting dissatisfaction with training; having chosen the teaching profession for job security; and complaining of difficulties with pupils and with administration.

A low score on the personal accomplishment subscale was significantly associated with female gender, being single, teaching in higher grade level (early secondary school, secondary school, vocational school and university), as well as reporting dissatisfaction with training and complaining of difficulties with pupils or with parents. Those who declared having chosen the teaching profession because they felt a calling for that were less susceptible to reduced personal accomplishment than those who did not. Particularly pronounced were the effects associated with the highest grade level taught, as compared with primary school.

## Discussion

This study on the personal and contextual factors associated with burnout in French teachers confirms a number of facts about burnout in this profession and also provides additional information about this syndrome. Our main finding relates to the multi-dimensionality of the burnout syndrome. Indeed, each of the three burnout dimension was associated with a specific set of covariates, and the effect of a given variable could possibly be in the opposite direction for different dimensions, as was the case for gender and grade level. This multi-dimensionality has been previously highlighted in teachers [11] as well as in diverse other professional groups [42], and should not be ignored when designing studies of burnout or intervention in that field.

Compared to various other studies of burnout in teachers in Europe, the mean dimension scores were essentially similar. However, it should be borne in mind that the study samples were not always comparable across studies. For instance, some included only secondary school teachers, one sample only primary teachers, one another only mathematics teachers. Given the importance of the population structure on the risk of burnout (with respect to gender and grade level repartition in particular), these figures have to be compared with caution. Our findings on burnout covariates cast new light on risk factors for burnout in teachers, complementing results obtained previously in other European studies [43].

In this study, gender was shown to be an important covariate of burnout, although the effect was not on the same direction for all dimensions. After adjustment for potential confounders (in particular, grade level), men were more susceptible to depersonalization, whereas women were more susceptible to emotional exhaustion and to reduced personal accomplishment. Gender differences in sources of stress and in social support may explain, in part, these observations [7]. Even when women hold outside employment (as was the case for female teachers in our sample), they still continue to take main responsibility for housework and children [44]. The combined demands of home and work life may result in higher level of psychological strain, whose long-term consequence could be a loss of energy (e.g. symptoms of emotional exhaustion) together with a withdrawal from work (and then a lower level of personal accomplishment). An element that may also contribute to gender differences in burnout symptoms is internalization of societal expectations. In particular, accepted norms associated with the male gender role emphasize strength, independence, professional achievement and invulnerability [45]. These may make men more likely to cope with their stress by developing dehumanized attitudes. In contrast, accepted norms associated with the female gender role emphasize softness and relationship. To cope with their stress without breaking these norms, women would be more susceptible to emotional exhaustion than depersonalization. On the whole, gender specificities in burnout were consistent with the sex differences in neuropsychiatric disease vulnerability, with women being at least twice as likely as men to suffer from depression and anxiety disorders [46].

Grade level was also shown in our study to be an essential associate of burnout. The representation of all grade levels in our dataset allowed us to evaluate the influence of this variable on the different dimensions of burnout. Emotional exhaustion scores were found to be higher in elementary school teachers than in teachers in early secondary schools, in secondary schools and universities. Conversely, depersonalization was associated with teaching in an early secondary school or, to a lesser extent, in a secondary school. Reduced personal accomplishment was also closely linked with teaching in secondary schools and in higher education. Difference in the work environment and in the sources of stress between grade levels (notably, pupils' attitudes and relationships with teachers, which evolve with age) is probably a key element underlying the contrasting pattern of associations between grade level and burnout dimensions.

The findings relating to teaching context provide novel information. In particular, teaching in an underprivileged area was associated with higher scores for both emotional exhaustion and depersonalization. The number of pupils taught was also associated with higher scores for emotional exhaustion. These contextual factors probably represent special additional challenges in teaching. Their importance for the risk of burnout appears particularly relevant to educational policy, since it is possible to control or change job-related conditions [11].

Burnout has seldom been considered in previous studies to be related to factors outside the work-worker interface, but in our study we found a significant association between marital status and burnout. Indeed, teachers who were single were more susceptible to both depersonalization and reduced personal accomplishment than those living with someone else. Such an association has been reported previously in the general population [47]. This observation supports the hypothesis of the family as a resource factor to cope with stress.

An interesting finding was that motivations for choosing the teaching profession were also related to burnout. Two completely different reasons were considered (job security and feeling a calling for teaching) and these influenced the different dimensions of burnout in opposite directions. Choice of teaching as a profession because of job security was associated with a higher risk of emotional exhaustion and depersonalization, whereas feeling a calling for teaching was linked with a decreased risk of reduced personal accomplishment. It should be noted that in a previous study of teachers carried out by the MGEN Foundation for Public Health [48], feeling a calling for teaching was associated with lower scores to the Ilfeld scale, which measured psychological stress. These results support the hypothesis that idealism in teachers may be a protective factor with respect to certain psychological symptoms. However, a wide gap between ideals and reality has also been shown to be a risk factor of burnout [49].

The present study has certain limitations. Firstly, it is vulnerable to selection bias because of its rather low response rate. As a consequence, representativity could not be ensured. However, the hypothesis that more heavily burdened teachers may be less likely to return their questionnaires is weakened by the fact that the scores on burnout dimensions observed in our final sample were quite similar to those obtained in a representative sample of Italian teachers [29]. It is thus likely that this potential selection bias is limited. As compared to the French population of teachers within the public education system, both younger teachers and university teachers were under-represented in the studied sample, but age and grade level were factored into our models as covariates, so this sub-optimal representativity should not influence the association findings. Secondly, the data were cross-sectional and collected from self-report. Reliance on self-reported data for the concomitant measurement of both burnout and its associates does not allow for causal conclusions. In particular, individuals with high negative affectivity may perceive their work context more negatively, which would strengthen artificially the associations between burnout symptoms and work environment. Longitudinal research is needed to identify causal determinants of burnout. A follow-up questionnaire is planned for 2010, and the data collected will be of great interest in this context.

In our study, outcomes of interest were based on the response to the MBI questionnaire. Other tools are available to measure burnout syndrome but not in French language. The French version of MBI has been previously validated in conditions that were rather similar to the present study (postal auto-questionnaire). In the validation study, indices of internal consistency, long-range stability, factorial validity, convergent validity and hypothetico-deductive validity support the general good psychometric properties of the instrument [28]. Cronbach's alphas evaluated in our sample of teachers supported further good internal consistency. Concerning covariates of interest, they were constructed on simple ad hoc questions. Response accuracy and reliability was supposed acceptable, but have not been specifically assessed.

Certain factors that have previously been considered important for burnout were not addressed by the questionnaire used in this study, in particular personality traits and individual resources. Nonetheless, we did consider certain perceptions and attitudes toward the socio-educational environment, namely dissatisfaction with certain aspects of teaching, reasons for choosing the teaching profession and difficulties in various spheres specifically related to the educational setting. We observed that such variables were closely linked with burnout dimensions. However, these associations have to be interpreted cautiously, because reversed causality cannot be excluded.

Our study was restricted to teachers, and the findings thus cannot be generalized to the whole working population, although they clearly have implications for the promotion of health and quality of life in teachers through reducing the risk of burnout. Studies on variables associated with burnout among teacher samples are scarce, particularly in France. To our knowledge, only one previous study has investigated cofactors of burnout among French teachers [29]. Our findings are consistent with this study, but given the much larger sample size, we were able to consider a much wider range of covariates, and to conduct adjusted multivariate analyses.

## Conclusion

Our study have highlighted the importance of contextual factors, namely: grade level, socio-professional neighbourhood and number of pupils, as potential determinants of burnout, supporting the possibility of social and organizational solutions in preventing and alleviating this syndrome. Our analyses confirm the multidimensionality of the burnout syndrome, which implies that interventions to reduce burnout should be planned and designed keeping in mind its different components. Longitudinal research is needed to explore the burnout mechanism in teachers further.

## Competing interests

The authors declare that they have no competing interests.

## Authors' contributions

VK and EN designed the study. FG performed the statistical analyses. PB and MNV drafted the manuscript. EN helped to draft manuscript. EN and MNV coordinated the study. MNV critically reviewed the manuscript and carried out all the revisions. All authors read and approved the final version of the manuscript.

## Pre-publication history

The pre-publication history for this paper can be accessed here:


